# Comparison of Surface Plasmon Resonance, Resonant Waveguide Grating Biosensing and Enzyme Linked Immunosorbent Assay (ELISA) in the Evaluation of a Dengue Virus Immunoassay

**DOI:** 10.3390/bios3030297

**Published:** 2013-07-31

**Authors:** Dongmei Hu, Scott R. Fry, Johnny X. Huang, Xixia Ding, Liwen Qiu, Yuxian Pan, Yue Chen, Jing Jin, Catriona McElnea, Joe Buechler, Xiaoyan Che, Matthew A. Cooper

**Affiliations:** 1 Division of Chemistry and Structural Biology, Institute for Molecular Bioscience, University of Queensland, Brisbane, 4072 Australia; E-Mails: zjyyhdm@163.com (D.H.); scottfry@optusnet.com.au (S.R.F.); j.huang2@uq.edu.au (J.X.H.); 2 Centre for Clinical Laboratory, Zhujiang Hospital, Southern Medical University, Guangzhou 510282, China; E-Mails: dxx53542008@126.com (X.D.); awen.qiu@163.com (L.Q.); pyx009@163.com (Y.P.); redcellchen@163.com (Y.C.); jingjin8211@yahoo.com.cn (J.J.); 3 Alere (Brisbane), 532 Seventeen Mile Rocks Road, Sinnamon Park, Qld, 4073 Australia; E-Mail: catriona.mcelnea@alere.com; 4 Alere (San Diego), Summer Ridge Road, San Diego, CA 92121, USA; E-Mail: joseph.buechler@alere.com

**Keywords:** Resonance Waveguide Grating, Surface Plasmon Resonance, dengue virus, label-free, diagnostic, affinity, kinetics

## Abstract

Two label-free biosensor platforms, Resonance Waveguide Grating (RWG) and Surface Plasmon Resonance (SPR), were used to rank a large panel of anti-dengue virus NS1 antibodies. Dengue non-structural 1 (NS1) protein is an established serological marker for the early detection of dengue infection. A variety of commercial dengue NS1 antigen capture immunoassays are available in both ELISA and lateral flow format. However, there is a significant scope to improve both the sensitivity and the specificity of those tests. The interactions of antibody (Ab)-antigen (Ag) were profiled, with weak interactions (K_D_= 1–0.1 μM) able to be detected under static equilibrium conditions by RWG, but not observed to under more rigorous flow conditions using SPR. There were significant differences in the absolute affinities determined by the two technologies, and there was a poor correlation between antibodies best ranked by RWG and the lower limit of detection (LLOD) found by ELISA. Hence, whilst high-throughput RWG can be useful as preliminary screening for higher affinity antibodies, care should be exercised in the assignation of quantitative values for affinity between different assay formats.

## 1. Introduction

Dengue is a major public health concern worldwide. It is estimated that more than 2.5 billion people live in endemic areas, with 50 to 100 million people suffering from dengue infection, resulting in approximately 25,000 deaths reported annually [1]. Despite efforts focused on vector control, the incidence of dengue has increased markedly over the past 50 years [2]. As a licensed vaccine or anti-viral therapeutics for dengue remain elusive, the control of dengue still relies heavily on an early and accurate diagnosis. Currently, the routine diagnostic methods of dengue include a combination of clinical signs, viral isolation [3], reverse transcription polymerase chain reaction (RT-PCR) [4], and serology [5]. Collectively these methods can provide accurate diagnosis; however, none of them are sufficiently sensitive and specific to be used as a stand-alone diagnostic tool [6].

Over the past decade, dengue virus (DENV) non-structural 1 (NS1) antigen capture immunoassays have been established and proven to be an effective tool in the diagnosis of dengue, particularly during the early stage of infection before the induction of a humoral immune response to the virus [7]. A number of DENV NS1 antigen capture immunoassays, mostly enzyme linked immunosorbent assay (ELISA) and immuno-chromatographic lateral flow assay (LFA) have been commercialized and are increasing in their acceptance and use globally [8,9,10]. However, the sensitivity of these approaches is limited by the infection status of patients [11], the virus serotype [12] and the assay performance [13]. On the basis of clinical evaluations of NS1 capture immunoassays across different endemic regions, the sensitivity of current tests can be considered adequate, but with significant scope for improvement [8,9], particularly for rapid and inexpensive lateral flow immuno-chromatographic tests.

To improve the sensitivity of a capture immunoassay, one critical factor is the efficacy of the capture moieties used, generally a non-competitive (matched) pair of bio-affinity molecules such as monoclonal antibodies (mAbs) or antibody fragments. Depending on the assay platform, the binding kinetics of the antibody-antigen can be tailored towards a high association rate (k_ass_), where the emphasis is on the rate of binding to maximize the number of antibody-antigen collisions during a short (e.g., the 5–10 min of a lateral flow assay) contact time, or a low dissociation rate (k_diss_) for assays which rely on equilibrium binding with high avidity (e.g., the hours needed for an ELISA assay with multiple washing steps).

Traditional immunologic techniques, such as ELISA, immunofluorescence assay (IFA) and Western blot (WB), detect molecular complexes using labeled secondary antibodies and are the most common means in the screening of antibodies. Although these are robust methods, they are time-consuming, labor-intensive and typically only provide limited information on the biophysical properties of antibody of interest. Moreover, the labeled reporter (fluorophore, luminophore, enzyme or radio-label) can sometimes alter the specific activity of the binding reagents and result in a loss of assay sensitivity and false negative results, or cause aggregation of protein, resulting in false positive results [14]. Label-free techniques overcome many of these assay limitations. Surface Plasmon Resonance (SPR) is an established label-free technique, which provides real-time information on binding rates of association and dissociation (kinetics), strength of an interaction (affinity), as well as the site of binding (epitope mapping) and determination of the active concentration of an antibody in solution [10]. It is an optical phenomenon that is sensitive to changes in the optical properties of the medium close to a metal surface. Currently, there are a few SPR systems available in the market, including BiaCore series, Biorad and Sierrasensors. In this study, we used the BiaCore T200 for all the SPR experiments. 

In addition to SPR, another label free technology, Resonant Waveguide (RWG) Grating, is widely used for large scale screening in recent years. The RWG sensors are composed of a periodic arrangement of dielectric material, in which a low refractive index periodic surface structure made of plastic is coated with a high refractive index film of Nb_2_O_5_ or TiO_2_. The reflected wavelength is changed by attaching biomolecules on the plate surface. A detection instrument illuminates the underside of the plate where the target material is loaded onto the bottom of the microplate well, and is capable of measuring all the sensors within one microplate in several seconds. Our strategy for efficiently ranking a large pool of dengue NS1 mAbs involved a preliminary screen using a benchtop 384-well EnSpire^®^ system (PerkinElmer), based on RWG technology [15] followed by detailed kinetic characterization by SPR and ELISA validation. A comparison of the three technologies has been performed. The pros and cons of each technology have been discussed. In addition, comprehensive binding properties of those mAbs have been acquired. 

## 2. Material and Methods

### 2.1. Materials

Anti-dengue NS1 mAbs were developed via traditional murine immunisation as described by Ding *et al.* [16]. Recombinant NS1 proteins were expressed in CHO cells [17]. An antibody cross-reactive with NS1 representing all four serotypes of dengue was provided by Alere, Australia, and was used as the reference antibody [17]. Polyclonal anti-dengue NS1 antibodies used in the ELISA lower limit of detection (LLOD) study were provided by Alere, Australia. Reagents for SPR assays including EDC (1-Ethyl-3-[3-dimethylaminopropyl]carbodiimde hydrochloride), NHS (N-hydroxysulfo-succinimide), ethanolamine, and mouse IgG antibody were purchased from Sigma-Aldrich (Sydney, Australia). 10× HBS-EP running buffer, 10 mM glycine pH 1.7, CM5 sensorchip and mouse antibody capture kit were purchased from GE Healthcare (Sydney, Australia). User-activated 384-well biochemical microplates were purchased from PerkinElmer (Melbourne, Australia).

### 2.2. Determination of Affinities of Anti-DENV NS1 mAbs Using a Resonant Waveguide Grating Biosensor

EnSpire^®^ user-activated biochemical plates (PerkinElmer Inc.) were activated with 15 μL of a 1:1 mixture of 400 mM EDC and 100 mM NHS in ultrapure water. The plate was incubated at room temperature for 30 min in the dark, followed by washing of the plate 3 times with ultrapure water using plate washer (BioTEK^®^, Millennium Science). Residual water was removed by centrifugation of the inverted plate for 1 min at 150 rcf. A 70 μg/mL solution of NS1 protein in 10 mM sodium acetate, pH 5.0 was added to each well followed by centrifugation at 150 rcf for 1 min. The plate was incubated at 2–8 °C overnight, and then washed 6 times with Phosphate Buffer Saline (PBS), pH 7.2. Unreacted sites of the well surface were quenched with 100 mM ethanolamine for 30 min, followed by a further washing with PBS. A baseline measurement was collected using the EnSpire^®^ Multimode Plate reader (PerkinElmer, Inc.) after a 2 h equilibration of the plate at room temperature. A 5-fold serial dilution series of anti-DENV NS1 mAbs from 2 μM to 0.257 nM was then transferred to the 384-well test plate in quadruplicate using a Biomek^®^ liquid handing system (Beckman Coulter). The plate was incubated at room temperature for 3 h and then a final series of 10 measurements were taken at 1 min intervals. In addition to blanks (no NS1), a well-characterized cross-serotype reactive NS1 antibody was included as a positive control and an irrelevant mouse IgG antibody was included as a negative control. Data was analyzed using the EnSpire^®^ label-free user interface software. The response values (pm) were plotted against the concentration of NS1 mAbs using GraphPad Prism^®^-5.0 software, with the (K_D_) determined by non-linear regression.

### 2.3. Kinetic Analysis of Interaction between NS1 Antigen and Antibody Using Surface Plasmon Resonance

All experiments were performed using a BiaCore T200 (GE Healthcare, Uppasala, Sweden) at 25 °C. All four flow cells of CM5 sensor chip were activated in parallel by a 7 min injection of freshly prepared 1:1 mixture of 400 mM EDC and 100 mM NHS at a flow rate of 10 μL/min, followed by 7 min immobilization of 30 μg/mL anti-mouse IgG in 10 mM acetate acid, pH 5.0 at flow rate of 30 μL/mL. Unreacted sites were blocked using 1 M ethanolamine-HCl PH 8.5 for 7 min at 10 μL/min. An immobilization level of 9,000 to 15,000 response units (RU) of anti-mouse IgG was obtained.

A multi-cycle kinetics method was used in which an optimized concentration of 2.5 nM of NS1 mAb was injected across the immobilized anti-mouse IgG surface at flow rate of 5 μL/min for 180 s to be captured on the CM5 chip. Then NS1 protein with the four-fold serial dilutions spanning 0.97 nM to 250 nM in HBS-EP running buffer, was injected serially in duplicate across the chip at a flow rate of 30 μL/min (association and dissociation time, 180 s, stabilization time, 60 s) with HBS-EP running buffer as the blank control. The chip surface was regenerated by an injection of 10 mM glycine-HCl, pH 1.7 at a flow rate of 30 μL/min for 40 s at the end of each cycle.

In this study, flow cells 1 and 3 served as the references for flow cells 2 and 4, respectively, to correct for bulk refractive index changes and non-specific binding. The sensorgrams were analyzed using BiaCore T200 evaluation software (version 1.0, GE Healthcare). Binding of NS1 mAb to all concentration series of NS1 protein was analyzed using a 1:1 binding model. The kinetic rate constants, association rate constant (k_ass_), dissociation rate constant (k_diss_) and equilibrium dissociation constant (K_D_), were calculated using the integrated evaluation software.

### 2.4. Determination of Dengue NS1 Capture ELISA LLOD

Selected mAbs, with SPR and RWG affinities comparable to Alere commercial monoclonal antibodies, were tested in a capture ELISA format. The ELISA format consisted of a polyclonal anti-dengue NS1 specific antibody coated onto a maxisorp plate (Thermo Fisher Scientific Cat# 446469) at 6 µg/mL in 50 mM carbonate buffer pH 9.8 and blocked with 2% BSA and 10% sucrose. Dengue NS1 antigen was captured by this polyclonal antibody. Four dengue NS1 serotypes were evaluated separately. Dengue NS1 antigen was titrated from 1,000 ng/mL to 0.009 ng/mL in four-fold serial dilutions. Selected anti-dengue NS1 mAbs, at a fixed concentration of 50 nM, were used to detect captured antigen. These mAbs were then detected with a cross-adsorbed anti-mouse IgG heavy and light chain peroxidase conjugate (Chemicon Cat# AP192P). Each plate included a negative control monoclonal antibody (non-reactive with NS1), a nil antigen control and the commercial Alere anti-dengue NS1 mAb as a positive control and a means of comparison between plates. Plates were incubated at 37 °C for 45 min each step. Six washes were performed between each step. Bound conjugate was detected using tetramethylbenzidine substrate (BioFx Cat# TMBW-1000-19) incubated at room temperature for 10 min, stopped with 1 M orthophosphoric acid and absorbance read at 450 nm wavelength.

### 2.5. Data Analysis

Data from RWG and SPR were plotted and statistically analyzed using GraphPad Prism^®^. The correlation of K_D_ value between RWG and SPR was measured by the Spearman test. The binding capacity of NS1 mAbs for NS1 protein, namely percentage activity in SPR were normalized against the amount of antibodies captured, was calculated as follows:

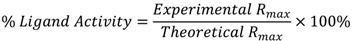

where the theoretical *R_max_* is determined from:

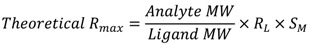

where *MW* is the molecular weight. *R_L_* (ligand response) is the amount of immobilized ligand in RU, and *S_M_* is the stoichiometry defined by the number of binding sits on the ligand. In this study, the analyte (NS1 proteins) *MW* was 300 kDa, the ligand *MW* (mAbs) was 150 kDa, the *S_M_* was revealed to be 1. The difference in percentage activity between RWG high- and middle-affinity NS1 mAbs (K_D_ = 10 nM and 100 nM, respectively) was compared using the Mann-Whitney test. 

Dengue NS1 capture ELISA LLOD was determined by the interpolation of best fit fourth order polynomial equations using GraphPad Prism^®^. LLOD was assumed to be three standard deviations above the absorbance for no antigen negative controls for each of the mAbs assessed. RWG and SPR affinity values were plotted against LLODs using GraphPad Prism^®^ and the Spearman correlation co-efficient determined.

## 3. Results

### 3.1. Characterization of Anti-Dengue NS1 mAbs

We developed 149 DENV NS1 mAbs using conventional hybridoma technique [16,18,19]. The immuno-reactivity of these mAbs with all four serotypes of DENV was characterized by ELISA and/or immunofluorescence assay (IFA) and/or Western blot (WB) [16,18,19]. In this study, NS1 mAbs that possessed immuno-reactivity in any of these three immunoassays were included in the RWG and SPR assays. Overall, there were 80, 55, 75, and 64 mAbs reacted with DENV-1, DENV-2, DENV-3 and DENV-4 NS1 respectively, with many antibodies possessing significant cross-reactivity (Table 1).

**Table 1:** Selectivity of dengue virus (DENV) reactive mAbs for each serotype used in this study.

Serotype origin of DENV NS1 mAbs	Number of DENV reactive mAbs characterized by immunoassays			
	DENV-1	DENV-2	DENV-3	DENV-4
DENV-1	32	1	12	8
DENV-2	17	31	21	21
DENV-3	20	14	28	11
DENV-4	11	7	14	24
Total	80	55	75	64

### 3.2. Affinity Measurements of DENV NS1 mAbs at Saturation Using RWG

**Figure 1:**
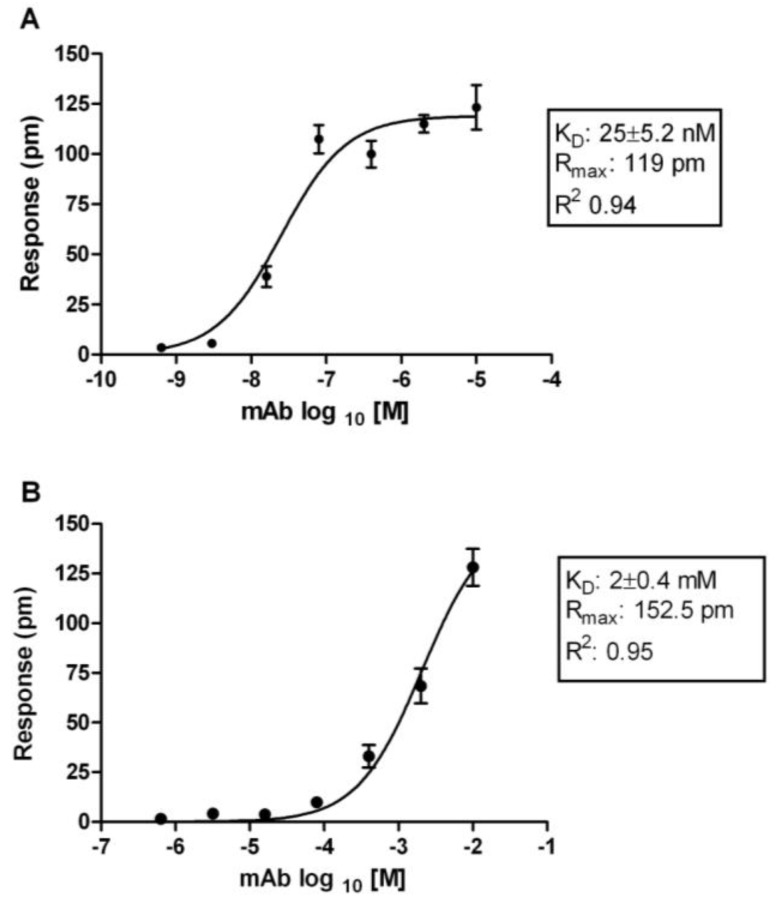
Representative binding isotherms for mAbs binding to non-structural 1 (NS1) measured by Resonance Waveguide Grating (RWG). (**A**) shows the saturated binding model of NS1 mAb (Dv1M1) to immobilized DENV NS1 protein, which generally produced middle to high affinity in RWG; (**B**) demonstrates the non-saturable binding model (Dv1 M4), which generally corresponded with low affinity. Values were given as mean ± standard error of the mean for n = 4.

**Figure 2:**
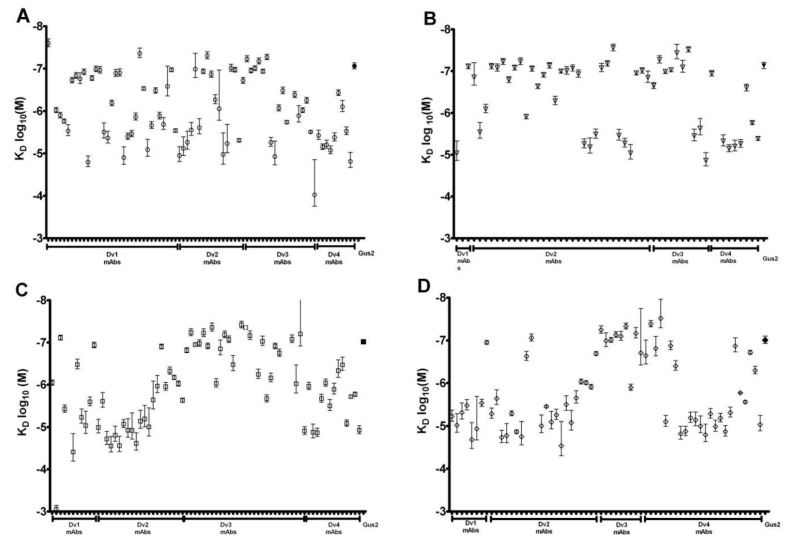
Affinities of DENV NS1 reactive mAbs detected by RWG. (**A**) DENV1 NS1 reactive mAbs, (**B**) DENV 2 NS1 reactive mAbs, (**C**) DENV3 NS1 reactive mAbs, and (**D**) DENV4 NS1 reactive mAbs. The affinity values (KD) of these NS1 mAbs ranged from 10^−8^ to 10^−3^ M. Error bars represent mean ± standard error of the mean for n = 4. The data point with solid fill is that obtained from the capture antibody (Gus 2) employed in the currently marketed Alere Dengue early diagnostic test.

RWG response values (picometer change in signal) of each mAb were plotted against the serial concentrations using GraphPad Prism^®^ software. The K_D_ values were calculated using a non-linear regression model as described in the methods section (Figure 1). On the basis of the plots, the calculated affinity, K_D_ values of these mAbs were determined (Figure 2) with a wide spectrum of affinity spanning nM to mM. In this study, mAbs with K_D_ ≥ 1 μM were determined as low affinity mAbs and not studied further (Table 2).

**Table 2:** The affinity range of DENV NS1 reactive mAbs measured by RWG (* Low affinity mAbs).

Rank of mAbs	Range of K_D_(M)	Number of DENV NS1 reactive mAbs in each serotype			
		DENV-1	DENV-2	DENV-3	DENV-4
High-affinity	10^−8^	8	19	12	9
Moderate-affinity	10^−7^	29	14	22	14
Low-affinity	10^−6^	29	17	21	26
	10^−5^	9	2	14	14
	10^−4^	1	0	1	0
Non-binders	<10^−3^ *	4	3	5	1
	Total	80	55	75	64

### 3.3. Binding Kinetics of NS1 mAbs Using SPR

**Figure 3:**
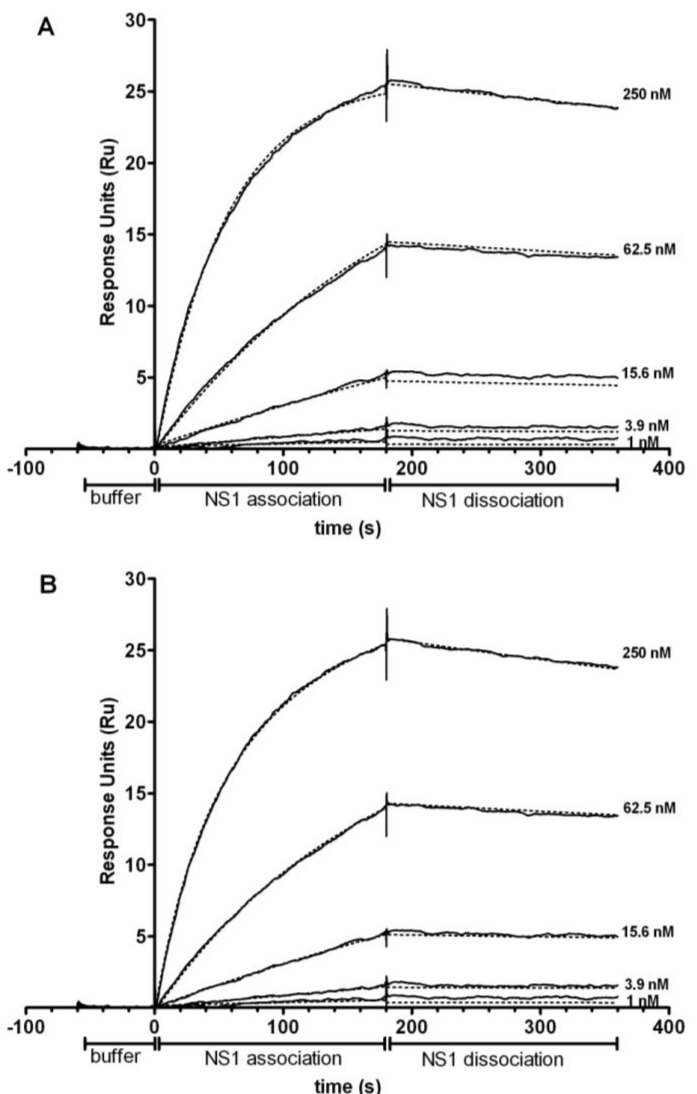
Representative sensorgrams of the interaction between injections of a DENV NS1 mAb (Dv3 M7) at varying concentration with immobilized DENV-3 NS1 protein. (**A**) Fitted as a 1:1 interaction model, (**B**) Fitted using a bivalent analyte model. The dotted lines represent the fitting of the original sensorgrams (blacklines).

**Figure 4:**
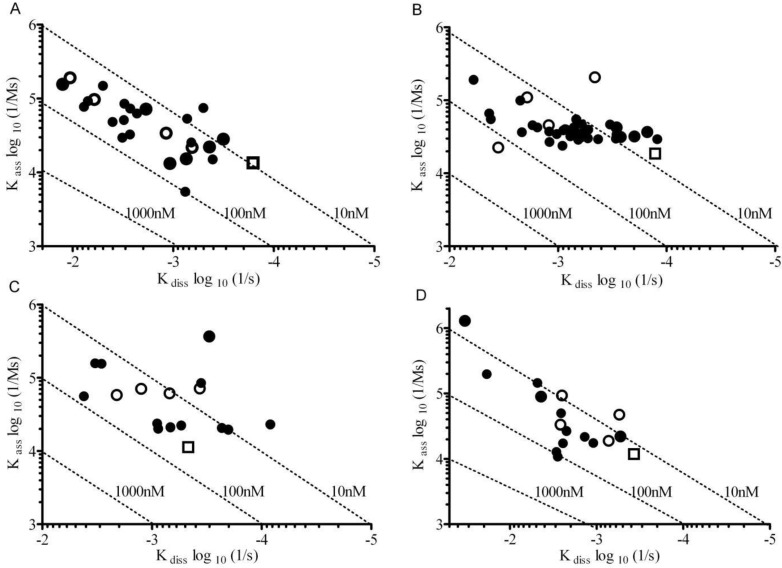
On-off rate plots of mAbs for DENV NS1 determined using Surface Plasmon Resonance (SPR).(**A**)DENV-1 NS1reactive mABs, (**B**) DENV-2 NS1 reactive mABs, (**C**) DENV-4 NS1 reactive mABs, and (**D**) DENV-4 NS1 reactive mABs. The dotted diagonal lines represent affinities (k_diss_/k_ass_). Open circles represent mAbs reactive with all four dengue NS1 serotypes. Open squares represent the commercial Alere mAb used for comparison.

The recombinant NS1 protein was in a predominantly hexameric structure, which was indicated by a Native-PAGE (data not shown). In addition, the mAbs have two binding sites against the NS1 antigen. As a result, a bivalent binding model should be more appropriate for data analysis. However, because of the two-step binding, the bivalent binding model will generate two K_D_ values [20], which make the ranking very complicated and almost impossible. Figure 3 shows an example (mAb Dv3 M7 binds to DENV-3 NS1) of the fitting results of the same sensorgrams using both 1:1 Langmuir binding model and bivalent binding model. The K_D_ values were calculated by k_diss_/k_ass_. In the 1:1 binding model, the K_D_ was 5 nM; whereas in the bivalent binding model, two sets of k_diss_ and k_ass_ were acquired, resulting in two K_D_ values (18 nM and 7.9 M). Considering the complication of bivalent model, we optimized the experimental conditions of SPR to promote the 1:1 binding in the flow system. These were achieved by capturing the lowest possible amount of mAb on the sensor chip surface and increasing the flow rate of sample injection to minimize avidity effects and mass-transport promoted re-binding. Therefore, a reasonable fitting was obtained (Figure 3(A)). The optimized SPR experiment hence measures the real kinetics of the interaction. The affinities of mAbs identified by SPR ranged from 1 nM to 200 nM (Figure 4). The k_ass_ ranged from 10^3^ to 10^5^ M^−1^·s^−1^, and k_diss_ ranged from 10^−7^ to 10^−2^ s^−1^.

### 3.4. Affinity Comparison of NS1 mAbs between RWG and SPR

The assignation of low-affinity interactions mAbs was consistent using either RWG or SPR, as mAbs with low-affinities determined by RWG displayed no response by SPR assay. Additionally, some RWG moderate-affinity mAbs also recorded no response in SPR (Table 3).

**Table 3:** Correlation of RWG and SPR affinities with enzyme linked immunosorbent assay (ELISA) lowerlimitofdetection (LLOD).

Dengue NS1 serotype	LLOD *vs*. RWG K_D_		LLOD *vs*. SPR K_D_	
	Correlation co-efficient (r^2^)	P value	Correlation co-efficient (r^2^)	P value
DENV-1	0.5506	0.0024	0.008603	0.8124
DENV-2	0.2804	0.2216	0.1409	0.4068
DENV-3	0.576	0.0479	0.3676	0.3937
DENV-4	0.07368	0.7286	0.03127	0.8232

**Figure 5:**
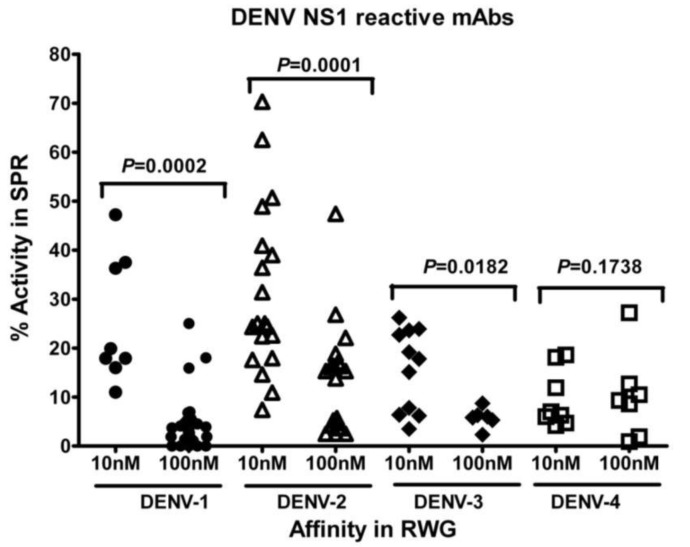
Comparison of SPR determined percent activity of mAbs betweenm Abs of different affinity subset, 10 nM and 100 nM in RWG. P value above the dots represents the statistical difference between both subsets of mAbs. In this study, except DENV-4 reactive mAbs, the percent activities of the other three mAb serotypes were significantly higher in mAbs with affinity of 10 nM (RWG) than that of with affinity of 100nM (RWG) using Mann-Whitney test.

For mAbs where affinities could be determined accurately by RWG *and* SPR, correlation analysis using the Spearman test demonstrated that DENV-1 (R = −0.1868, P = 0.3507) and DENV-3 (R = −0.05147, P = 0.8445) NS1 reactive mAbs RWG and SPR affinities were not correlated. However, DENV-2 (R = 0.3656, P = 0.0394) and DENV-4 (R = 0.7559, P = 0.0007) NS1 reactive mAbs displayed a moderate correlation. The percentage activities of the antibodies (the amount determined by comparison of the theoretical and experimental maximal response; see Section 2.5) determined by SPR and RWG were correlated within different affinity subsets. The Mann-Whitney test demonstrated that the percentage activities of DENV-1 (U = 10, P = 0.0002), DENV-2 (U = 47, P = 0.0003) and DENV-3 (U = 9, P = 0.0182) NS1 reactive mAbs were significantly higher in the RWG high-affinity mAbs subsets than that of RWG middle-affinity mAbs. Although the difference of percentage activity of DENV-4 reactive mAbs was not so significant (U = 18, P = 0.1738), they displayed the same trend (Figure 5).

### 3.5. Correlation of SPR and RWG Affinity with Capture ELISA LLOD

There was a poor correlation between affinity values derived by SPR or RWG, and ELISA LLOD (Table 4).

**Table 4:** Comparison of ELISA LLODs and affinities of selected anti-dengue NS1 MAbs.

MAb ID	Serotype specificity	LLOD (pg/mL)	RWG K_D _(nM)	SPR K_D _(nM)
Dv1 M32	1	4.1	44	37
Dv1 M19	1	4.2	103	36
Dv1 M1	1	4.3	25	37
Dv3 M7	1	4.5	54	7
Gus 2	1	4.7	87	12
Dv3 M18	1	4.9	414	27
Dv2 M27	1	5.6	137	83
Dv3 M4	1	6.8	99	61
Dv1 M33	1	3.0	296	11
Dv2 M4	2	3.0	77	23
Dv2 M27	2	3.2	28	7
Dv2 M10	2	3.2	60	12
Gus 2	2	3.2	75	7
Dv2 M17	2	3.4	101	38
Dv2 M14	2	3.8	124	16
Dv3 M4	2	4.1	94	27
Gus2	3	4.7	96	42
Dv3 M11	3	4.7	86	10
Dv3 M7	3	5.4	44	5
Dv3 M4	3	4.2	105	11
Dv4 M27	4	4.2	506	141
Dv3 M7	4	4.4	47	12
Dv4 M6	4	5.0	134	33
Gus 2	4	5.5	99	31
Dv3 M4	4	4.1	99	38

## 4. Conclusion and Discussion

Dengue NS1 immunoassays can be used to diagnose dengue infection early in the course of disease [21]. Compared to virus isolation and RT-PCR methods, they offer a cheap and convenient option that only requires basic technical training. However, clinical studies indicate that commercially available dengue NS1 detection assays have poor sensitivity, influenced by serotype and geographical variability of isolates [22,23]. Indeed sensitivities as low as 40% were recorded in one study [22]! There is considerable scope to improve sensitivity by selection of appropriate capture and detection antibodies with higher affinity, better binding kinetics and improved recognition of dengue NS1 across all serotypes.

In an effort to improve the throughput for evaluating of DENV NS1 antigen capture immunoassays, two optical label-free biosensor techniques, RWG and SPR, were used to screen a collection of DENV NS1 mAbs. RWG is a high-throughput method, which can detect up to as many as 40,000 molecular interactions within 8 h [15]. Therefore, we used RWG as a preliminary affinity screen to rank antibodies. A broad range of affinities from mM to nM were observed. However, when these mAbs were characterized by SPR using a 1:1 Langmuir binding kinetics model only antibodies with an apparent RWG K_D_ in the nM range produced dose-dependent responses.

The lack of correlation between the affinities determined by RWG and SPR may be attributable to several factors. First of all, the K_D_ values acquired from the RWG study were based on steady state analysis; whereas those from SPR were calculated using a kinetics model based on 1:1 binding. Although a steady state model can be achieved in SPR, it requires much higher concentrations and flow rates that result in the consumption of a large amount of materials. Secondly, the RWG study used antigen bound to a plate coated with proprietary maleic anhydride polymer binding chemistry, while the SPR study uses captured antibodies on a hydrophilic carboxymethyl dextran polymer layer with NS1 binding from free solution. In SPR, the rebinding seems to be a major factor affecting the accuracy of SPR-based kinetics for antibody profiling. Although we optimized the SPR running condition to improve 1:1 binding, the presence of rebinding in SPR can’t be entirely avoided. It can systematically increase the apparent binding affinity by decreasing the dissociation constant measured. In contrast, RWG only measures the equilibrium binding affinity, which is much simpler to evaluate than a flow-based system. Thirdly, several studies have demonstrated that some anti-NS1 mAbs recognize linear epitopes while others only conformational epitopes [24,25,26], hence the surface chemistry in the biosensor systems must be able to maintain the native structure of the antigen. Immobilization of NS1 may possibly impact its conformation and presentation of epitopes. The arrangement of the NS1 hexamer, as determined by single particle analysis, indicates that some epitopes may remain hidden and that antibody binding can trigger a change in conformation of the hexamer [26]. Alteration in the conformation of the NS1 may affect the affinity with which antibodies bind to their target for both linear and conformation epitopes and may in part explain the variation seen in affinities recorded between RWG and SPR assays. Thus, whilst RWG is useful as a first pass screening tool, care should be exercised in the assignment of absolute affinities. Thirdly, one should establish a screening method closely resembles the conditions that will be used in the diagnostic immunoassays. Although SPR is now a standard method for measuring the affinity of antigen-antibody interactions, careful experimental design and data processing are necessary to determine accurate rate and affinity constants [27]. 

This was also borne out in the poor correlation between SPR and RWG affinity data with the limit of detection found in ELISA format. It is possible that this may in part be due to the different surface chemistries utilized for binding ligands. RWG used amine linkage to maleic anhydride polymer, SPR used amine linkage to a carboxymethylated dextran surface while ELISA used hydrophobic interaction with a polystyrene surface. The assay formats also varied, with RWG using fixed antigen, while SPR and ELISA used antigen in solution. RWG and ELISA measurements are carried out under equilibrium conditions (a static system) with a long contact time while SPR used a flow cell with limited contact time. There was a broad rank order correlation with mAbs showing less than 10^−7^ M affinity by either SPR or RWG showing no detectable signal by ELISA.

Our results indicate that RWG can be used for broad screening to discriminate between good and poor binders; however, detailed information regarding binding kinetics is lacking. The SPR instrument (BiaCore T200) used in this study is not as high through put as the RWG equipment. Nevertheless, it gave accurate kinetics parameters such as k_ass_ and k_diss_ that are necessary for future application of the mAbs. The combination of RWG, SPR and ELISA can provide all the information needed for the design of much more sensitive and specific immunoassays. 
